# Dietary Exposure of Pacific Oyster (*Crassostrea gigas*) Larvae to Compromised Microalgae Results in Impaired Fitness and Microbiome Shift

**DOI:** 10.3389/fmicb.2021.706214

**Published:** 2021-08-24

**Authors:** Julien Vignier, Olivier Laroche, Anne Rolton, Pandora Wadsworth, Karthiga Kumanan, Branwen Trochel, Xavier Pochon, Nick King

**Affiliations:** ^1^Aquaculture Group, Cawthron Institute, Nelson, New Zealand; ^2^Coastal and Freshwater Group, Cawthron Institute, Nelson, New Zealand; ^3^Moana New Zealand Ltd., Nelson, New Zealand; ^4^Institute of Marine Science, The University of Auckland, Warkworth, New Zealand

**Keywords:** oyster hatchery, larvae, *Crassostrea gigas*, microalgae, fitness, microbiome, 16S rRNA gene sequencing

## Abstract

The Pacific oyster *Crassostrea gigas* is the world’s most cultivated oyster and seed supply is heavily reliant on hatchery production where recurring mass mortality events are a major constraint. Outbreaks of bacterial infection *via* microalgal feed are frequently implicated in these mortalities. This study assessed the effects of feeding compromised microalgae to developing oyster larvae. Intentionally ‘stressed’ (high pH) or non-stressed microalgae were fed to 11 day-old oyster larvae at two feeding rations for 96 h, followed by a recovery period. Biological endpoints of larval performance were measured following the 96 h exposure and subsequent recovery. Bacterial communities associated with the microalgae feed, rearing seawater, and the oyster larvae, were characterized and correlated with effects on oyster fitness parameters. Feeding stressed algae to oyster larvae for 96 h increased the occurrence of deformities (>70% vs. 20% in control), reduced feeding and swimming ability, and slowed development. Following the recovery period, fewer larvae reached pediveliger stage (2.7% vs. 36% in control) and became spat (1.5% vs. 6.6% in control). The quantity of stressed algae supplied to oyster larvae also influenced overall larval performance, with high feeding rations generally causing greater impairment than low rations. Bacterial profiling using 16S rRNA showed that most bacterial families characterized in larval tissue were also present in larval rearing seawater and in the microalgae feed (98%). The rearing seawater showed the highest bacterial richness compared to the larval and the microalgal compartments, regardless of feeding regime. In larval tissue, bacterial richness was highest in stressed and high-feed treatments, and negatively correlated with larval fitness parameters. These results suggest significant dysbiosis induced by compromised feed and/or increased feed ration. Several bacterial genera (e.g., *Halomonas, Marinomonas*) were strongly associated with impaired larval performance while the presence of genera in larvae including *Vibrio* was closely associated with overfeeding. Our research demonstrated that metabarcoding can be effectively used to identify microbiota features associated with larval fitness.

## Introduction

The Pacific oyster, *Crassostrea gigas*, is a major commercial species and the most cultivated oyster globally with a production value estimated at US$ 1.24 billion p.a. (Houston et al., 2020). Hatchery production of *C. gigas* is currently the only sustainable way of reliably supplying this significant industry but the recurrence of mass mortality events in hatcheries is a major constraint to increasing spat supply and enabling sustained productivity. These mortality events are generally linked to outbreaks of bacterial infections (Tubiash et al., 1965; Elston and Leibovitz, 1980; Nicolas et al., 1996; Prado et al., 2005). Prophylactic measures to reduce such mortality events include; enhanced biosecurity and hatchery hygiene, improved husbandry (e.g., reduction of stocking density, temperature, increase of water renewal), the use of antibiotics or probiotics and routine water monitoring (Sindermann and Lightner, 1988; Prieur et al., 1990).

Marine microalgae produced by mass-culture are an essential food source for many bivalve species cultured in hatcheries, including *C. gigas*, and a reliable supply of ‘good quality’ algae is paramount for optimal spat output (Loosanoff and Davis, 1963; Walne and Spencer, 1974; Helm and Millican, 1977; Coutteau and Sorgeloos, 1992; Helm and Bourne, 2004).

Bivalve larvae fed ‘good quality’ algae display rapid growth, early settlement, and high metamorphic rates (Holland and Spencer, 1973; Robert and Gérard, 1999; Powell et al., 2002; Robert et al., 2017). A microalgal diet rich in lipids has been shown to be particularly essential at the late larval stage of *C. gigas* when feeding requirements and growth are at their highest (Powell et al., 2002; Rico-Villa et al., 2009; Ben Kheder et al., 2010). Conversely, larval stages of bivalves fed with “poor quality” or compromised algal cultures may be adversely affected, resulting in developmental delays and reduced overall fitness (Ragg et al., 2010; Kaspar et al., 2014). In addition, adapting the quantity (or ration) of algal food to the nutritional requirements of the developing larvae is challenging, and overfeeding larvae, especially with compromised algae, may exacerbate the negative effects on larval settlement and reduce oyster spat production (Powell et al., 2002; Rico-Villa et al., 2009). Traditional quality assessments of algal cultures generally consist of daily visual checks, cell counts, water quality monitoring, and bacterial community monitoring by selective cultivation. Such assessments are tedious and unlikely to detect problems with algal health in a timely manner, increasing the risk of feeding compromised algae to larvae for several days prior to detection. In a recent study by Rolton et al. (2020), in which two commercially important species for aquaculture, *Tisochrysis lutea* (T-Iso) and *Chaetoceros calcitrans*, were reared in a stressful environment by intentionally subjecting the cultures to high-pH conditions, Pulse Amplitude Modulation (PAM) fluorometry and flow cytometry (FCM) were successfully used to identify a variety of indicators of algal function, including photosynthetic parameters and morphological changes. This allowed earlier detection of ‘compromised’ algae compared to traditional algal health measurements (Rolton et al., 2020). In the same study, the bacterial abundance and populations were measured using FCM, showing increased bacterial density per algal cell for both algal species and higher bacterial diversity (T-Iso) in the high pH treatment compared to control, hence providing valuable information on the condition of algal cultures during periods of relatively mild stress (Rolton et al., 2020).

Despite precautionary measures to maintain microalgal cultures free of contamination from bacteria, large scale monospecific algal cultures grown in the hatchery are generally non-axenic (Nicolas et al., 2004). The presence and role of bacteria both within algal and larval cultures is complex (Nicolas et al., 2004; Powell et al., 2013; Laroche et al., 2018; Arfken et al., 2021). Certain bacterial species may be considered beneficial within algal cultures, through the production of vitamins and other growth promoters (Haines and Guillard, 1974; Suminto and Hirayama, 1997; Le Chevanton et al., 2013) and have demonstrated probiotic effects when added to bivalve larval cultures (Douillet and Langdon, 1994; Prado et al., 2010; Kesarcodi-Watson et al., 2012; Stevick et al., 2019). Conversely, other bacterial species can affect bivalve cultures in the hatchery by reducing larval motility and feeding, leading to larval growth retardation and ultimately failure of the whole larval batch (Walne, 1956; Nicolas et al., 1996; Prado et al., 2005). Microalgae fed to larvae are generally seen as the most probable introductory source of these pathogenic bacteria, notably due to the proliferation of opportunistic bacteria in the nutrient-enriched algae growing medium (Nicolas et al., 1989; 2004; Saulnier et al., 2009; Dubert et al., 2015). Pathogenic *Vibrio* spp. have been commonly implicated in bivalve hatchery outbreaks of bacterial disease (Elston and Leibovitz, 1980; Nicolas et al., 1996; Prado et al., 2005, 2014; Elston et al., 2008; Rojas et al., 2016); however, since these bacteria are often present in association with healthy larvae, it is not clear how pathogenicity is initiated (Elston et al., 2008).

Until recently, research into hatchery epizootics has been largely limited to studying the culturable bacterial populations associated with larval cultures of bivalves, with *Vibrio* and *Pseudomonas* spp. being the most frequently isolated organisms (Kueh and Chan, 1985; Prieur et al., 1990). Selective culturing has been shown to represent only 0.01–12.5% of the viable bacterial population in a marine environment (Ferguson et al., 1984). Consequently, bacteria implicated in hatchery outbreaks/epizootics are likely to be vastly underestimated.

Recent developments in high-throughput sequencing technologies combined with the use of 16S rRNA metabarcoding have enabled complete microbiome characterization of environmental and tissue samples in an extremely time and cost-effective manner (Caporaso et al., 2011; Taberlet et al., 2012). Several studies have taken advantage of the resolution and scalability offered by environmental DNA metabarcoding (herein intra- and extra-cellular DNA from water and tissue) to characterize microbiomes associated with adult oysters (King et al., 2012; Trabal et al., 2012; Wegner et al., 2013; Trabal Fernández et al., 2014; Lokmer and Wegner, 2015; Simons et al., 2018) and to study host-pathogen interactions (e.g., POMS; De Lorgeril et al., 2018; King et al., 2019a,b,c; Lasa et al., 2019; Clerissi et al., 2020). Few studies have investigated microbiomes associated with oyster larvae in the hatchery (Powell et al., 2013; Asmani et al., 2016; Laroche et al., 2018; Stevick et al., 2019; Arfken et al., 2021) and to our knowledge, none have investigated their microbial community turnover under different feeding regimes and culture conditions.

The aims of this study were to (i) evaluate the potential effects of feeding compromised microalgae to early life stages of Pacific oysters by assessing their larval performance and spat yield; (ii) characterize and compare the bacterial communities associated with the microalgal feed, oyster larvae and the seawater in which larvae were reared, and (iii) identify microbiota features associated with reduced larval development and yield.

## Materials and Methods

### Experimental Animal Production

#### Broodstock Conditioning, Spawning, and Incubation

Twenty-four adult Pacific oysters, *Crassostrea gigas*, of wild origin were conditioned for 10 weeks at 22°C (± 1) and fed *ad libitum* with a mixture of naturally occurring phytoplankton species bloomed by fertilized eutrophic ponds and continuously cultured monospecific algae (*Tisochrysis lutea*, CS-177, and *Chaetoceros muelleri*, CS-176). On 15th of July 2019, oysters were strip-spawned according to Allen and Bushek (1992), and gametes from 8 ripe females and 3 males were kept for fertilization. Approximately 600 million eggs were obtained, gently washed through a 50 and 15 μm screen mesh, and then fertilized at a sperm to egg ratio of 200:1. Following fertilization, embryos were incubated at a stocking density of 250 embryos mL^–1^ in static 170-L tanks previously filled with 12 μM EDTA-treated seawater adjusted at a temperature and pH of 25°C and 8.4, respectively.

#### Larval Rearing System and Procedure

At 1-day post-fertilization (or Day 1 PF), veliger D-larvae were collected and stocked in a 170-L cylindro-conical fiberglass tank (=stock tank) at a starting density of 250 larvae mL^–1^. Filtered seawater (FSW), consisting of previously carbon and mechanically filtered (1 μm), UV-treated (100 mJ/cm^2^) seawater, was adjusted to 24°C and supplied continuously to the larval rearing tank at a flow rate of ∼3 L min^–1^. Larvae were fed a bispecific algal diet of *Chaetoceros calcitrans* (CS-178) and *Tisochrysis lutea* (T-*Iso*) throughout rearing. Algal cultures were supplied to the larval rearing tank by computer-controlled pneumatic pumps via feeding lines (Ragg et al., 2010). Algal concentration was maintained at appropriate density and feeding rates throughout larval rearing. The appropriate number of algal cells to feed larvae each day was based on the larval consumption of algae from the previous day. Algal density was determined using a Coulter Counter (Beckman Coulter Multisizer 4 particle analyzer, Beckman Coulter Inc., California; 2.5–20 μm, according to Ragg et al., 2010).

Oyster larvae were reared under these conditions during which time no significant mortality was reported. At day 11, oyster larvae were collected for use in the experimental assay.

### Experimental Design

#### Preparation of Algal Treatments

Batch cultures of *Chaetoceros calcitrans* (CC) and *Tisochrysis lutea* (T-Iso) were grown according to Rolton et al. (2020). Briefly, carboys for both algal species were prepared with 18 L of 0.35 μm FSW to which double strength F2 culture medium (Cell Hi F2P, Varicon Aqua Solutions Ltd., Worcester, United Kingdom) and Si (for CC only) were added. Carboys were autoclaved and 500 mL inoculum cultures were added to each of the 18 L carboys. Inoculated carboys were supplied with 3 L min^–1^ aeration with added CO_2_ (0.3% v/v) through a 0.2 μm filter and were maintained at 21°C and exposed to continuous light at ∼200 μmol photons m^–2^ sec^–1^ (Kaspar et al., 2014). ‘Stressed’ microalgal cultures were created by removing the CO_2_ input to the carboys 24 h after algal inoculation, to induce high pH conditions. Algae from the non-stressed (or Control) and stressed carboys were fed to the oyster larvae after 5 days of culture for CC and 7 days of culture for T-Iso (Rolton et al., 2020).

To confirm that algal cultures were effectively stressed, cell density, pH and several indicators of algal photosynthetic parameters were measured by Pulse Amplitude Modulation (PAM) fluorometry and Flow Cytometry (FCM) before algal cultures were fed to the larvae (see “Measurements of Algal Parameters”). New carboys of CC and T-Iso were grown each day and stressed independently.

Stressed and control microalgae were fed daily at a 60:40 ratio (CC: T-Iso) to oyster larvae at two different rations: a low ration of 25 cells (equivalent T-Iso) per μL, referred to as “Control Low” or “Stress Low,” and a high ration of 100 cells per μL referred to as “Control High” or “Stress High” throughout. The daily volumes of algal feed to support optimal growth and metamorphosis rates were calculated using the following formula, based on Helm and Bourne (2004):

Volumetofeed(mL)=[requiredcelldensity(cellsperμL)×

Volumeoftank(mL)]/celldensityofharvestedalgae(cellsperμL)

#### Exposure of Larvae to Stressed Microalgae and Subsequent Recovery

##### Exposure of larvae

**FIGURE 1:**
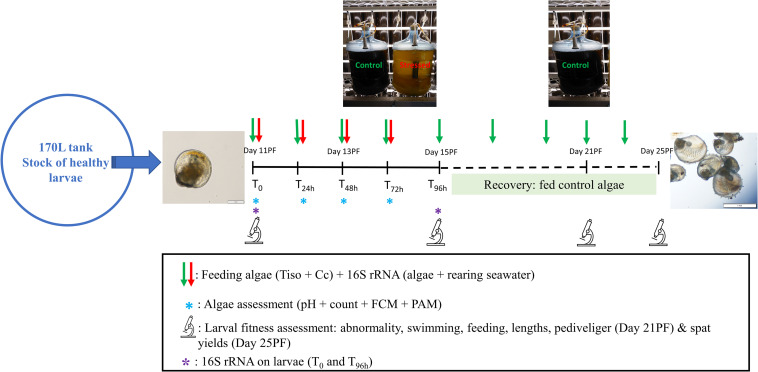
Experimental design. Tiso, *Tisochrysis lutea*; Cc, *Chaetoceros calcitrans*; PF, post-fertilization; FCM, flow cytometry; PAM, pulse amplitude modulation; 16S rRNA, microbiome metabarcoding.

Eleven day-old or Day 11 PF (i.e., late umbo stage, mean shell length of 164 μm ± 2.4), oyster larvae were collected from the stock tank, counted and distributed in six replicate 2.5 L purpose-built polycarbonate larval rearing tanks at a density of 5 larvae mL^–1^ (or 12,500 larvae per tank) (Figure 1). Larvae were reared at 23°C (± 1) using a static renewal system and fed for 96 h with either stressed or control algae at a low and high ration. A complete water exchange was carried out at 48 h, during which larvae were washed on a separate 90 μm screen (one per treatment) to retain all larvae (live and dead). At 96 h (or Day 15), a final assessment was conducted followed by screening/grading of the larvae on 150 μm mesh.

##### Recovery of larvae

Following the final 96 h assessment from the larval exposure, larvae from all treatments were returned to their respective tanks, fed a normal ration (i.e., 40 cells equivalent T-Iso per μL) of control algae and complete water exchanges combined with grading (using increasing screen size) were performed every other day until larvae developed to the spat stage (Figure 1).

### Sampling and Assessment

#### Measurements of Algal Parameters

Carboys of stressed and control CC and T- Iso were fed to the oyster larvae when the algal cultures were 5 days old and 7 days old, respectively. On T_0_, T_24 h_, T_48 h_, and T_72 h_ of the larval exposure assay, approximately 50 mL from non-stressed/control and stressed cultures of CC and T-Iso were collected before feeding the larvae to determine a) pH, b) microalgal cell concentration, c) photosynthetic, and d) physiological parameters (Figure 1).

a. The pH was measured using a handheld Testo 206 pH meter.
b. Microalgal cell concentration was estimated by counting Lugol-fixed algal cells in five random 1 mm^2^ squares of a 0.1 mm-deep Neubauer hemocytometer (Weber Scientific International, Hawksley Technology, England). The mean of the five counts was calculated and results are presented as cells mL^–1^.
c. Photosynthetic parameters were determined using Pulse Amplitude Modulation (PAM) fluorescence analyzer (Aquapen AP 110-C). Light-adapted quantum yield (Fv’/Fm’), corresponding to the photosystem II (PSII) photosynthetic efficiency of algae in the light, and the fluorescence (Ft) of the algae were characterized before each feeding. The instrument was used on default settings with a 455 nm wavelength measuring flash pulse of 0.03 μmol m^–2^ sec^–1^ and a saturating pulse 2100 μmol photons m^–2^ sec^–1^ (Rolton et al., 2020).
d. Morphological and physiological parameters of algal cells were analyzed using a FACSCalibur flow cytometer (BD Biosciences, San Jose, CA, United States) equipped with a 488 nm (blue) argon laser and three fluorescence detectors, green (FL1, 530 nm), orange (FL2, 585 nm), and red (FL3, > 670 nm). Incubation of samples with fluorescent dyes was performed in the dark at room temperature (∼20°C) prior to flow cytometric analysis. Unless stated, results are expressed in arbitrary units (a.u.).

Cellular assays, extensively described in Rolton et al. (2020), assessed the: relative size (Forward Scatter, FSC) and complexity (Side Scatter, SSC) of the algae, the chlorophyll content (red fluorescence, FL3), cell viability (SYTOX^TM^ Green, S7020, Invitrogen for CC, and Fluorescein diacetate FDA, F1303, Invitrogen for T-Iso), reactive oxygen species (ROS) production (DCFH-DA, CAS No. 4091-99-0, Sigma Aldrich^®^) and neutral lipid content (BODIPY^TM^ 493/503, D3922, Molecular probes). Data were analyzed using ‘Cell Quest Pro’ software.

#### Assessment of Oyster Larval Performance

##### After 96 h exposure to compromised microalgae

Larval fitness parameters were estimated from three subsamples, each containing 100 individuals, collected directly from the top of each tank, and assessed under magnification (×10) after fixation with 10% buffered formalin. The final number of larvae at the end of the 96 h assay (corresponding to Day 15 PF larvae) was estimated by first concentrating the larvae from each tank into a 500 mL beaker, and then counting the number of live and dead larvae (exhibiting translucent shells, opened valves, or no internal organization).

**FIGURE 2:**
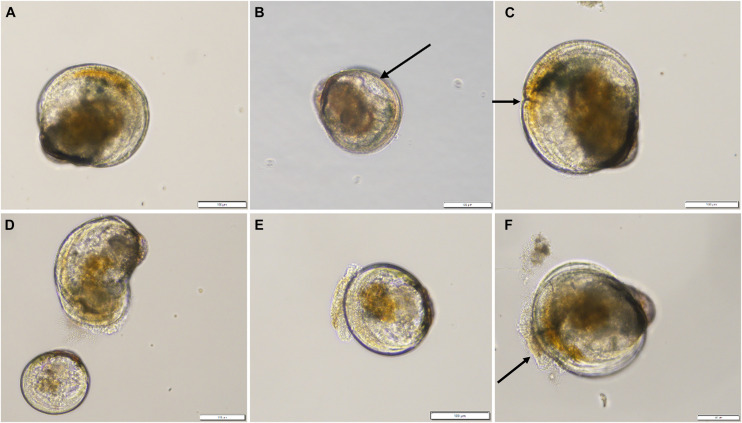
Examples of normal **(A)** and abnormal **(B–F)** *Crassostrea gigas* larvae observed in the “Stressed low” and “Stressed high” treatments after 96 h. Anomalies consisted of indented mantle (arrow, **B**), indented shell margin (arrow, **C**), severe shell deformity (**D** top), pale non-feeding larvae **(D)**, protruded velum **(E)**, or abnormal presence of bacteria on velum (arrow, **F**). Scale bar: 100 μm.

The percent of normal (Figure 2A) and abnormal larvae from each subsample were assessed on Day 15 (T_96 h_) according to His et al. (1997). Larval abnormalities included: indented mantle (Figure 2B), indented shell margin (Figure 2C), deformed shell (Figure 2D), protruded velum (Figure 2E) and abnormal presence of bacteria (Figure 2F).

The percent of feeding larvae was determined from the same sample by counting the number of larvae with algal food in the stomach (brown coloration) and the number of pale larvae with no food in the stomach (e.g., Figure 2D).

Larval swimming activity was evaluated from each tank on Day 15 (T_96h_) by counting the number of resting or inactive larvae versus the total number of larvae (inactive and actively swimming) 1 min after swirling in a tissue culture dish (TCD) containing a non-fixed sample. Larvae swimming in arcing trajectories were considered ‘actively swimming.’ Spinning behavior by larvae was considered abnormal swimming and those individuals were included with inactive/resting larvae (McDougall et al., 2020).

The shell lengths of 30 larvae per tank were randomly measured on Day 11 (T_0_), and Day 15 (T_96h_), using CellSens image analysis software (inverted microscope CX41 20 × magnification, DP74 camera; Olympus).

##### After the recovery period

Following the recovery phase (section “Recovery of larvae”), the pediveliger success and spat yield were determined on Day 21 PF (after 7 days in ‘recovery phase’) and Day 25 PF (after 10 days in ‘recovery phase’), respectively (Figure 1). On Day 21 PF, the content of each tank was poured through a 235 μm screen and the number of larvae which had reached pediveliger stage (defined as the presence of an eye spot) were counted. When larvae reached competency (i.e., development of eyespot, gills, production of mucus, and crawling behavior), Epinephrine at 10^–4^ M (CAS No 51-43-4; Sigma Aldrich^®^, United States) was added to promote larval settlement and metamorphosis (Coon et al., 1985), larvae were rinsed after 1 h and returned to the tanks.

At Day 25 PF, the content of each tank was poured through a 275 μm screen and spat retained on the screen were resuspended in a 1 L beaker. After thorough mixing, 5 aliquots were sampled from the beaker and enumerated. Pediveliger success and spat yield were calculated based on the number of larvae initially stocked on T_0_ of the assay (corresponding to Day 11 PF).

### Microbiomic Analyses

#### Sampling

One-liter samples of FSW were collected from the influent water line (supplied to the larval rearing tanks) on T_0_, T_24h_, T_48h_, and T_72h_ (or Day 11, 12, 13, and 14 PF), using gloves and a sterile (autoclaved) Schott glass bottle. Additionally, one-liter composite samples of FSW medium used to prepare algal cultures (post-autoclave) were collected periodically before inoculation of the carboys with algae. The content of each bottle was then filtered through a sterile 47 mm membrane cellulose filter of 0.22 μm pore size (Whatman), and the membrane filter was then placed in a sterile, DNAse-free microtube and stored at −80°C until DNA extraction. Up to 50 mL samples of each species of algae (CC and T-Iso) were collected from stressed and control carboys on Day 11, 12, 13, and 14 PF, and filtered following the same procedure as described for seawater. Approximately 1000 oysters were collected from the control and stressed treatments for each ration (i.e., low and high), at the start (T_0_/Day 11) and end (T_96h_/Day 15) of the larval exposure (Figure 1). Larval samples were washed thoroughly with R.O water on a 55 μm screen and placed in a microtube using a disposable plastic pipette. Supernatant was then removed after decantation and samples were stored at −80°C until extraction.

#### DNA Extraction, PCR Analyses, and Sequencing

A total of 52 samples were processed for total DNA extraction, including 6 seawater samples, 30 oyster larval samples, and 16 algal samples. Additionally, four blank DNA extractions were included in order to test for potential bacterial contamination of the DNA extraction kit and/or reagents. Samples were homogenized via bead beating for 10 min at 1500 rpm using a 1600 MiniG automated tissue homogenizer (SPEX Sample Prep, Metuchen, NJ, United States; 1600 MiniG Spex SamplePrep, NJ, United States), and centrifuged (10,000 rpm, 1 min, 20°C; Eppendorf Centrifuge 5430R, Hamburg, Germany). The supernatant (ca. 500 μL) was transferred into a new tube and DNA was extracted using the Qiagen DNeasy PowerSoil kit (Qiagen, CA, United States), all according to the manufacturer instructions. The quantity and quality of extracted DNA were measured using a NanoPhotometer (Implen, Munich, Germany).

Polymerase chain reactions (PCR) were performed on all extracted samples (*n* = 52, plus blank controls). Bacterial communities were amplified using the 16S rRNA gene (v3–v4 region) with the primer set 341F: 5′-CCT ACG GGN GGC WGC AG-3′ (Herlemann et al., 2011) and 805R: 5′-GAC TAC HVG GGT ATC TAA TCC-3′ (Klindworth et al., 2013). These primers were modified to include Illumina^TM^ overhang adaptors (forward: 5′-TCG TCG GCA GCG TCA GAT GTG TAT AAG AGA CAG-3′ and reverse: 5′- GTC TCG TGG GCT CGG AGA TGT GTA TAA GAG ACA G-3′), as described in Kozich et al. (2013). To reduce the amplification of microalgal chloroplast DNA, blocking primers were designed as described in Laroche et al. (2018). Briefly, two blocking primers were incorporated to specifically target *Chaetoceros* (5′-TCT AAT CCC ATT TGC TAC CCT A-C3-3′) and *Tisochrysis* (5′- TCT AAT CCC TTT TGC TAC CCT A-C3-3′) species. The designed blocking primers contained at least five mismatches with bacterial 16S rRNA sequences and annealed to the microalgal template at the 3′-end of the 805-R reverse primer. The blocking primers also included a C3 spacer at the 3′-end so that extension of the PCR product did not occur. The blocking primers were included at 10 times the concentration of universal 16S rRNA primers during PCR amplification.

Polymerase Chain Reactions were carried out in 40 μL reaction volumes containing 20 μL MyFi 2 × PCR supermix (Bioline, London, United Kingdom), 15 μL of nuclease-free H_2_0, 0.20 μM of modified Illumina overhang adaptor primers, 2.0 μM of both blocking primers, and 2 μL of template DNA. Thermocycling conditions were: 94°C for 3 min, followed by 38 cycles of 94°C for 30 s, 52°C for 30 s, 72°C for 1 min, with a final extension step at 72°C for 5 min.

Amplicons were purified using the SequalPrep^TM^ normalization plate kit (Applied Biosystems^TM^, CA, United States) resulting in an equimolar concentration of ∼1 ng μL^–1^, all according to the manufacturer’s instructions. Purified amplicons were individually indexed using the Nextera^TM^ DNA library Prep Kit (Illumina, CA, United States) and paired-end sequenced on a MiSeq Illumina^TM^ with the 2 × 250 bp v2 kit at New Zealand Genomics Ltd (NZGL, Auckland, New Zealand). Raw sequence data is publicly available in the NCBI database under project number PRJNA729567.

### Bioinformatic Analysis

Demultiplexed fastq files were quality filtered and denoised in R with the DADA2 program (Callahan et al., 2016), using the default parameters when not specified otherwise. Prior to merging with a minimum overlapping region of 8 bp, the forward and reverse reads were trimmed at 17 and 21 bp of their 5′ end respectively, to remove primers, and truncated at 243 and 241 bp respectively to increase the yield of good quality denoised reads. *De novo* chimera detection was performed using the DADA2 consensus approach where sequences found to be chimeric in the majority of samples are discarded. Taxonomic assignment was performed with the RDP Naive Bayes Classifier algorithm (Wang et al., 2007) implemented in DADA2, and trained on the SILVA 16S rRNA database (release 132 clustered at 99% similarity; Quast et al., 2012). Non-bacterial sequences or those assigned to chloroplast were removed from the dataset. Sequence reads found in the blank samples (DNA extraction, PCR, indexing) were removed from the dataset using the MicroDecon R package (McKnight et al., 2019). To reduce noise from rare taxa or possible library artifacts, amplicon sequence variants (ASVs) with less than 2 reads in a minimum of 3 samples were discarded. Sampling depth per sample was visualized with the ‘rarecurve’ function of the ‘vegan’ R package (Oksanen et al., 2019), and following previous recommendations (Caporaso et al., 2011), samples with insufficient sequence coverage (less than 5,000 reads) were removed from downstream analyses.

### Statistical Analyses

Statistical analyses of algal and larval fitness parameters were performed using SigmaPlot 14.0 (SYSTAT Software, Inc.). All percentage data were arcsine square root transformed to improve normality. Normality of data distribution was tested using the Shapiro–Wilk test (*p* < 0.05) while homogeneity of variances was checked using the Brown–Forsythe test (*p* < 0.05). After fulfilment of these two conditions, Student’s *t*-tests were employed to identify significant differences of algal parameters, under control and stress conditions, whereas one-way analysis of variances (ANOVA) were conducted to compare measurements of larval performance for each treatment. When significant effects of treatments were found for ANOVA (*p* < 0.05), multiple comparisons between the means using a Tukey *post hoc* test were conducted.

Microbial taxonomic composition was visualized with bar plots using the ‘phyloseq’ R package (McMurdie and Holmes, 2013) and bacterial ASVs richness between datasets (algae, rearing seawater and larvae) compared with box plots on data rarefied to lowest sequencing depth among samples (i.e., 5,162 reads). Additionally, differences in larvae microbiome richness between treatments was visualized with box plot and tested with a linear mixed model (lmm) using the ‘lme4’ R package (version 1.1.27; Bates et al., 2015; formula = log richness ∼ algae stress treatment ^∗^ ration, with tank as random effect), and results reported with the ‘report’ R package (version 0.3.5, Makowski et al., 2020). Associations between bacterial richness and fitness metrics were assessed with Pearson correlations using the ‘chart.Correlation’ function of the ‘PerformanceAnalytics’ (version 2.0.4) R package, with richness log transformed, and fitness parameters transformed to the square root to improve the normal distribution of the data. Whenever possible, analyses of the microbial data were performed using a Compositional Data Analysis (CoDA) approach as advocated in Calle (2019). As such, Beta-diversity analyses (bacterial taxa turnover between tanks) were performed using the Aitchison distance by creating Euclidean distance matrices on centered-log ratio transformed (clr) abundance data with the ‘microbiome’ R package (Lahti and Shetty, 2019). Ordinations were visualized with principal component plots (PCAs) using the ‘phyloseq’ package; the 15 families correlating the most to sample ordination were determined via the envfit function of the ‘vegan’ R package (Oksanen et al., 2019), and overlaid as vectors. In addition, correlation between larval fitness measurements and bacterial communities were assessed using the envfit function and visualized by overlaying the parameters as vectors in the PCAs. Differences in community diversity were assessed with permutational analyses of variance (PERMANOVA) with treatment crossed with days of exposure using the ‘adonis2’ function of ‘vegan.’ Within group dispersions were assessed with the ‘betadisper’ function of the ‘vegan’ package. The proportion of bacterial genera shared between the larvae, algae and seawater datasets was investigated and visualized with a Venn diagram using the ‘eulerr’ R package (Larsson, 2019). Identification of taxa strongly affected or associated with the algae treatment was performed using the ANCOM methodology (Mandal et al., 2015) implemented in Qiime2 (Boylen et al., 2018), and visualized using the ‘ggplot2’ R package (Wickham, 2016).

## Results

### Algal Parameters

Before cultures of *C. calcitrans* (CC) and *T. lutea* (T-Iso) were fed to the experimental oyster larvae each day, a suite of parameters was measured to verify the cultures were effectively stressed by the high-pH conditions (Figure 1). The pH in the ‘Stressed’ cultures was significantly higher than in the non-stressed or control cultures of CC (8.98 vs. 7.5, *p* < 0.001) and T-Iso (8.75 vs. 7.6, *p* < 0.001), respectively (Table 1). Algal cell densities were significantly lower in the stressed cultures than in the control cultures of CC (5.7 × 10^6^ vs. 24 × 10^6^, *p* = 0.013) and T-Iso (2.4 × 10^6^ vs. 5.8 × 10^6^, *p* = 0.002) (Table 1). The light adapted quantum yield and fluorescence as determined by PAM fluorometry were also significantly lower in the stressed algal treatments compared to control cultures (0.52 vs. 0.72 and 5729 vs. 2093 for CC and 0.72 vs. 0.62 and 3870 vs. 1453 for T-Iso respectively, Table 1). Differences in the algal morphology, chlorophyll content, ROS production, viability and neutral lipid content as measured by flow-cytometry were also observed in the stressed cultures of CC and to a lesser extent, cultures of T-Iso, compared to controls (Table 1).

**TABLE 1:** Algal health parameters of *Chaetoceros calcitrans* (CC) and *Tisochrysis lutea* (T-Iso) cultures which had been ‘stressed’ under high pH conditions or, were non-stressed (Control) before feeding to oyster larvae.

	CC		T-Iso	
	Control	Stressed	Control	Stressed
pH	7.5	8.98***	7.6	8.75***
	(± 0.1)	(± 0.16)	(± 0.14)	(± 0.12)
Cell density (10^6^ cells mL^–1^)	24	5.7*	5.8	2.4**
	(± 3.6)	(± 0.64)	(± 0.6)	(± 1.2)
Quantum yield (F_*v*_’/F_*m*_’)	0.72	0.52***	0.72	0.62***
	(± 0.01)	(± 0.03)	(± 0.01)	(± 0.01)
Fluorescence (Ft)	5729	2093**	3870	1453*
	(± 638)	(± 343)	(± 618)	(± 98)
Size (a.u.)	45.51	80.55*	99.36	115.68
	(± 2.37)	(± 8.32)	(± 7.63)	(± 7.55)
Complexity (a.u.)	9.48	35.19**	16.83	18.22^#^
	(± 0.30)	(± 4.65)	(± 0.25)	(± 0.52)
Chlorophyll FL3 (a.u.)	14.18	19.13***	23.55	21.53
	(± 0.48)	(± 0.38)	(± 1.05)	(± 1.14)
ROS production (a.u.)	4.73	30.75^#^	12.82	12.38
	(± 1.34)	(± 18.59)	(± 0.72)	(± 0.32)
Viability (%)	99.86	89.42*	98.96	97.86
	(± 0.06)	(± 3.97)	(± 0.35)	(± 0.7)
Neutral Lipid (a.u.)	4.08	22.34*	17.89	16.75
	(± 0.12)	(± 3.60)	(± 1.15)	(± 0.84)

Results are expressed as the mean (± SEM) of 4 carboys using time as replication (i.e., day 0, 1, 2, and 3). Asterisks indicate significant effect of treatment at: ^#^p < 0.1; *p < 0.05; **p < 0.01; ***p < 0.001 using t-tests. ROS, reactive oxygen species; a.u., arbitrary units.

### Effects of Compromised Algal Feed on Larval Fitness Parameters

#### Larval Abnormalities, Feeding, Swimming, and Shell Length After 96 h Exposure

**FIGURE 3:**
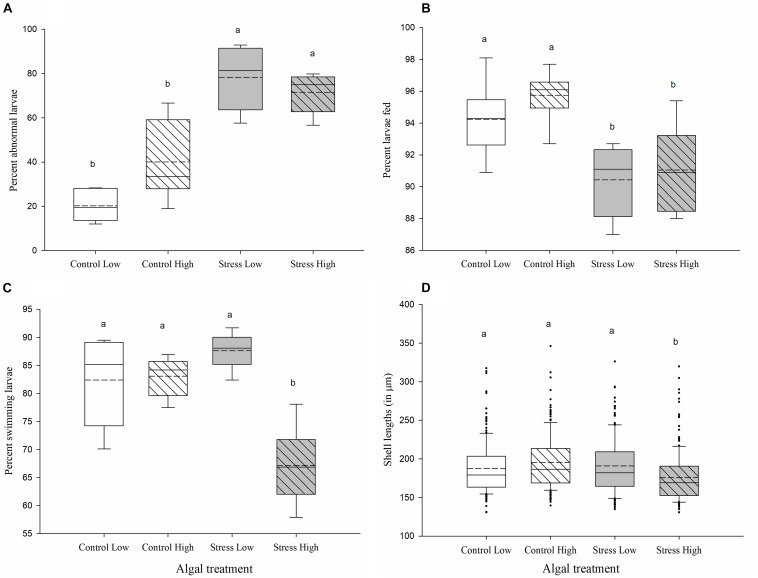
Measurements of larval performance in *Crassostrea gigas* exposed to control and stressed algal cultures for 96 h. Percentages of **(A)** larval abnormalities, **(B)** feeding larvae, **(C)** actively swimming larvae, and **(D)** shell lengths of larvae (in μm) were evaluated following 96 h of exposure to control/unstressed algae (white), and stressed algae (gray). Low ration (plain box) consisted of 25 cells μL^−1^ of a bispecific diet of *C. calcitrans* (CC) and *T. lutea* (T-Iso), while high ration (hashed box) consisted of 100 cells μL^−1^ of CC and T-Iso. A total of 100 larvae were assessed for abnormality and 30 larvae for feeding and swimming response from each tank. Box plots represent the distribution of the fitness parameters, in percent, between the sixtanks **(A–C)** or the shell sizes of *n* = 180 larvae **(D)**, per algal treatment. Median and mean (dashed line) values and the lower (25%) and upper (75%) quartiles are presented, while whiskers indicate the minimum and maximum values, and individual points are outliers (**D** only). Treatments with the same letter were not significantly different (*p* > 0.05; ANOVA, Tukey *posthoc* test).

More abnormalities were recorded in larval cultures which had been fed stressed algae at both high and low rations for 96 h (78.4% ± 5.9 and 71.7% ± 3.7, respectively), compared to larvae in the control treatments (40% ± 7.3 in Control High and 20.2% ± 2.8 in Control Low, *p* ≤ 0.05, Figure 3A). Most abnormalities recorded were shell deformities, extrusion of the mantle, abnormally extended velum and incomplete mantle (Figure 2).

Exposing larvae to stressed algal cultures for 96 h also negatively affected the larval feeding, swimming activity and shell lengths. Fewer larvae fed with either low or high ration of stressed algae had food present in their stomach compared with larvae fed with either the low or high ration of control algae (90.4% ± 0.9 and 91.1% ± 1.1 in the Stress Low and Stress High treatment vs. 94.3% ± 1 and 95.7% ± 0.7 in the Control Low and Control High treatments, *p* ≤ 0.05, Figure 3B). Moreover, higher instances of larvae exhibiting signs of “constipation” (aggregated algal cells could be observed attached to the shell hinge) were recorded in both the Stress Low and Stress High algal treatments. Finally, additional microscopic observation indicated a reduced number of lipid droplets, particularly in the body cavity of larvae in the Stress High treatment (data not shown).

The swimming activity of larvae fed the high ration of stressed algae for 96 h was significantly lower than in all other treatments (*p* ≤ 0.001). Only 67.5% (± 2.8) exhibited normal swimming activity with the remainder showing increased lethargic/static and spinning behaviors (Figure 3C). Over 83% of larvae in the control treatments, regardless of algal ration, and the Stress Low treatment, showed normal swimming activity (Figure 3C).

Larvae in the Stress High treatment were significantly smaller after 96 h exposure (169 μm ± 1.5) than those in the Control Low (179 μm ± 3), the Stress Low (181.9 μm ± 2.4), and the Control High treatments (186.6 μm ± 1.8) (*p* < 0.05; Figure 3D).

#### Pediveliger Success and Spat Yield After Recovery

**FIGURE 4:**
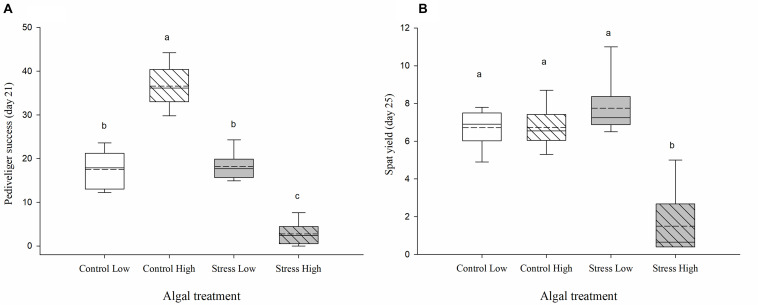
**(A)** Pediveliger success and **(B)** final spat yield, expressed as percent, assessed before settlement on day 21 PF and day 25 PF, respectively. *Crassostrea gigas* larvae were exposed for 96 h to control/non-stressed algae (white), and stressed algae (gray) and subsequently reared for 7–10 days under normal feeding ration of unstressed algae (i.e., recovery conditions). Low ration (plain box) consisted of 25 cells μL^−1^ of a bispecific diet of *C. calcitrans* (CC) and *T. lutea* (T-Iso), while high ration (hashed box) consisted of 100 cells μL^−1^ of CC and T-Iso. Box plots represent the distribution of the pediveliger success **(A)** and spat yield (B), in percent, between the sixreplicated tanks per algal treatment. Median and mean (dashed line) values and the lower (25%) and upper (75%) quartiles are presented, while whiskers indicate the minimum and maximum values. Pediveliger success and spat yields were based on the number of umbo larvae estimated at test initiation on day 11 PF. Different letters indicate significant differences (*p* ≤ 0.05) between each group (ANOVA, Tukey *posthoc* test).

Assessment of pediveliger success and spat yield during the recovery phase (during which larvae were fed a normal ration of control algae and were maintained under optimal husbandry conditions) occurred after 7 and 10 days, respectively. Larvae that had been exposed for 96 h from 11 to 15 days PF to stressed and control algae prior to the recovery phase, showed contrasting development (Figure 4). The highest percentage of pediveliger success was in the Control High treatment (36.2% ± 2, Figure 4A). By comparison, only 2.7% (± 1.1) of the larvae previously exposed to the Stress High treatment reached the pediveliger stage (*p* < 0.001; Figure 4A) and 17.6% (± 1.7) and 18% (± 1.4) of larvae reached the pediveliger stage in the Control Low and Stress Low treatments, respectively (*p* ≤ 0.001, Figure 4A).

Finally, the lowest spat yield was obtained in tanks previously exposed to the Stress High algal treatment with only 1.5% (± 0.75) of the larvae metamorphosing into spat, compared to 6.7% ± 0.4 and 6.6% ± 0.5 in the Control Low and Control High treatments and 7.7% ± 0.7 Stress Low treatment (*p* ≤ 0.001, Figure 4B).

### Characterization of Bacterial Communities

A total 10,493,536 reads (mean of 145,744 per sample) were sequenced, of which 78% remained after quality filtering, denoising and chimera removal. Removal of sequences found in negative controls and those associated to Chloroplast or non-bacterial taxa further reduced the number of reads by 6 and 23%, respectively. Removing taxa with less than 2 reads in at least 3 samples reduced reads by 5%, leaving a mean of 62,116 reads per sample. Five samples with low read counts (< 5,000 reads) did not have sufficient sequencing depth to reach complete diversity coverage and were removed from downstream analysis.

#### Composition of Bacterial Assemblages Amongst the Three Compartments

**FIGURE 5:**
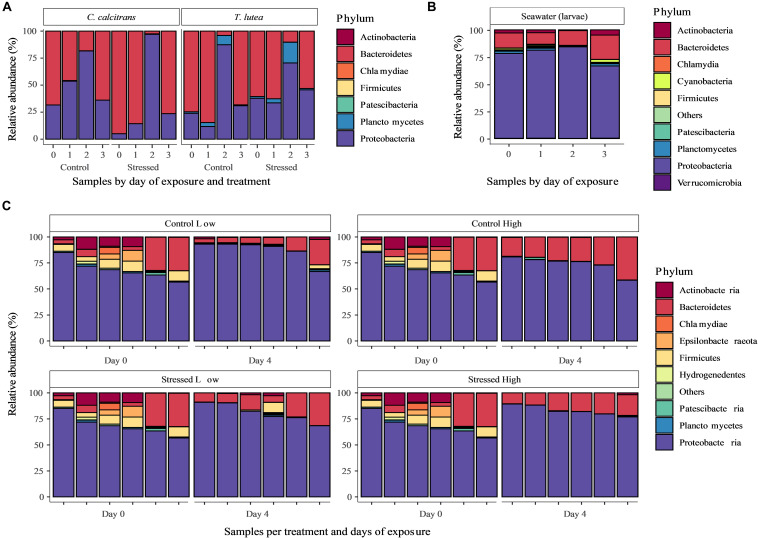
The taxonomic composition, expressed as relative abundance in %, of microbiomes associated with **(A)** *Chaetoceros calcitrans* (CC) and *Tisochrysis lutea* (T-Iso) cultures on each day of the 96 h larval exposure under control and stressed (high pH) conditions; **(B)** the seawater in which oyster larvae were reared, on each day of the 96 h exposure; and **(C)** Pacific oyster (*Crassostrea gigas*) larvae at the start (Day 0) and at the end of the exposure (Day 4) to a low and high ration of unstressed microalgae (Control Low and Control High) and compromised algae (Stressed Low and Stressed High). Approximately 1000 larvae were collected from each tank on Day 0 and Day 4 (96 h) of the assay; each bar represents one tank (*n* = 6); larval microbiome on Day 0 was quantified from one pool of healthy control larvae that were subsequently distributed across the four treatments: as a result, “Day 0” data arerepeated and presented four times to allow clearer comparison with “Day 4” data.

Similar bacterial phyla were recorded in the 3 compartments (microalgal feed, seawater in which larvae were reared and oyster larvae), although their relative abundance varied. The bacterial taxa identified in samples of both microalgal species were almost exclusively composed of *Bacteroidetes* and *Proteobacteria* with variations in relative abundance between days of exposure (Figure 5A). A small fraction (<20%) of *Planctomycetes* were also present in cultures of T-Iso on two of the 4 days of the experimental exposure. No discernable effect of pH stress on the composition of bacterial taxa at the phylum level could be observed (Figure 5A).

The bacterial taxa identified in the seawater samples used for larval rearing were primarily composed of *Proteobacteria* (>65%), followed by *Bacteroidetes* (∼15%) and *Actinobacteria* (∼3%), with proportions relatively constant across days of exposure (Figure 5B).

The bacterial taxa associated with larvae were also primarily composed of *Proteobacteria* (>60%), followed by *Bacteroidetes* (∼15%) and, at the start of exposure, *Actinobacteria* (∼3%). *Epsilonbacteraeota* and *Firmicutes* were noticeably more present at the start of the exposure period (Figure 5C). At phylum level, no discernible effect of treatment could be observed on the composition of bacterial taxa associated with larvae.

#### Bacterial Diversity and Commonality Between Compartments

**FIGURE 6:**
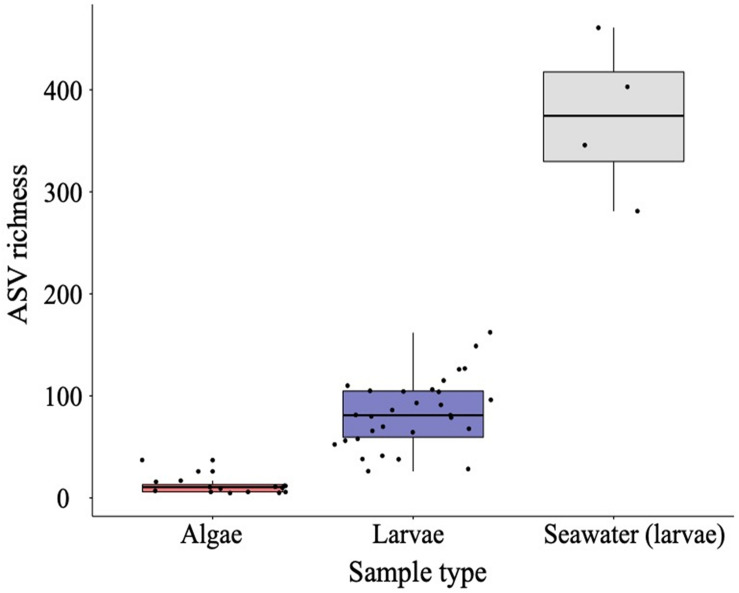
Boxplots of amplicon sequence variant (ASV) bacterial richness evaluated in *Chaetoceros calcitrans* (CC) and *Tisochrysis lutea* (T-Iso) microalgae, *Crassostrea gigas* larvae, and rearing seawater datasets. All treatments and days of sampling data were pooled. Median values and the lower (25%) and upper (75%) quartiles are presented. Whiskers indicate the minimum and maximum values, while outliers are displayed as dots.

The highest bacterial richness (at ASV level) was recorded in rearing seawater (mean ± SD, 373 ± 77), followed by larval oyster samples (83 ± 34), and microalgal samples (12 ± 8) (Figure 6). While most bacterial families were shared across all compartments, the rearing seawater samples had a non-negligible proportion of unique taxa (8% of total families; Supplementary Figure 1). Only 2 families from the microalgal samples were not present in the larval samples. Families of particular interest including *Flavobacteriaceae, Rhodobacteraceae*, and *Vibrionaceae* were found in all compartments while *Halomonadaceae* was present in all but algae seawater samples, and *Marinomonadaceae* unique to the larvae compartment (Supplementary Figure 1).

#### Bacteria Associated With Oyster Larvae

To allow a direct comparison with larval fitness parameters, further analysis of bacterial richness was done on larval samples only.

**FIGURE 7:**
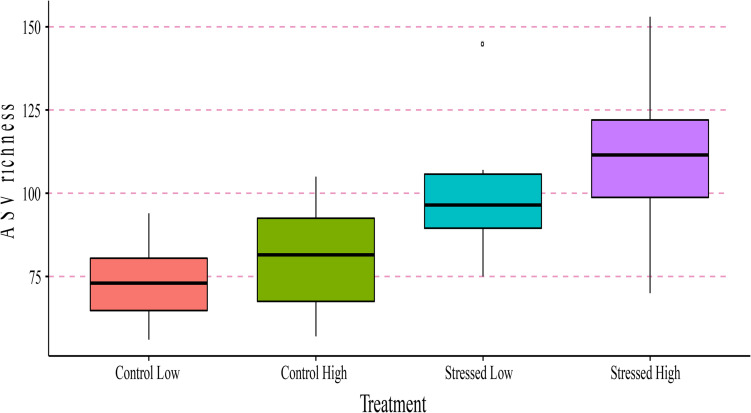
Boxplots of amplicon sequence variant (ASV) richness evaluated in *Crassostrea gigas* larvae after 96 h (day 15 PF) per treatment condition (Control Low, Control High, Stress Low, and Stress High). Median values and the lower (25%) and upper (75%) quartiles are presented. Whiskers indicate the minimum and maximum values, while outliers are displayed as dots.

We fitted a linear mixed model (estimated using REML and nloptwrap optimizer) to test the effect of algae treatment and ration on log transformed bacterial richness (formula: log(richness) ∼ algae treatment ^∗^ ration ^∗^ 1| tank). The model’s explanatory power related to the fixed effects alone (marginal *R*^2^) was 0.35. Within this model: the effect of stressed algae was statistically significant and positive (β = 0.32, 95% CI [0.06, 0.57], *t*(18) = 2.40, *p* = 0.017; Std. β = 1.17, 95% CI [0.21, 2.13]; Figure 7). However, the effect of high ration and it’s interaction with algae treatment were found non-significant. Standardized parameters were obtained by fitting the model on a standardized version of the dataset, and 95% Confidence Intervals (CIs) and *p*-values computed using the Wald approximation.

Interestingly, no difference in bacterial richness could be observed between control and stressed cultures of CC and T-Iso samples (Supplementary Figure 2).

Bacterial richness in the larvae showed a significant and strong positive correlation with the larval mortality rate (*r* = 0.6, *p*-value = 0.002) and abnormality rate (*r* = 0.45, *p*-value = 0.027) at 96 h, and a significant negative relationship with pediveliger success (*r* = −0.45, *p*-value = 0.026) after the recovery period (Supplementary Figure 3).

##### Bacterial beta-diversity

**FIGURE 8:**
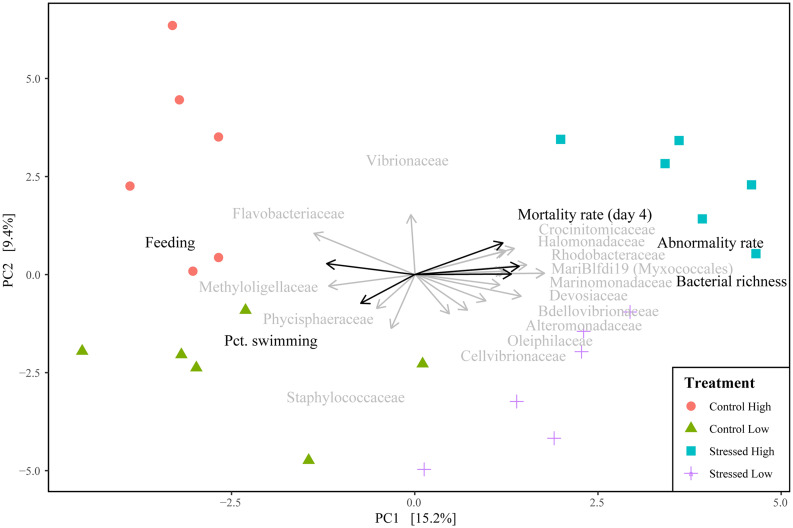
Principal components analysis (PCA) of community composition of *Crassostrea gigas* larvae after 96 h of exposure to stressed algae per treatment condition (Control Low, Control High, Stress Low, and Stress High). The 15 most important families are displayed as vectors (gray arrows) using the mean score value of their associated ASVs.

Principal component analysis (PCA) ordination of the larval dataset showed strong clustering per treatment, with greater mortality rate, abnormality rate and bacterial richness in the “Stressed High” treatment (Figure 8). In contrast, larval feeding and swimming rates were positively associated with the “Control High and Low” treatments, respectively. Permutational analysis of variance showed a significant effect of exposure duration (*R*^2^ = 0.136, *p* = 0.001), as well as pH stress (*R*^2^ = 0.099, *p* = 0.001) and feeding ration (*R*^2^ = 0.06, *p* = 0.005, Supplementary Table 1). The effects of pH treatment (*R*^2^ = 0.134, *p* = 0.001) and algal feeding ration (*R*^2^ = 0.082, *p* = 0.002) were also significant in samples of larvae which had been exposed to compromised algae for 96 h (Table 2); however, their interactions were non-significant (*p* = 0.138; Table 2).

**TABLE 2:** Permutational analysis of variance of centered-log transformed microbiome diversity based on Euclidean distance matrix between treatment conditions of samples at day 4 of exposure using 999 permutations.

Terms	Df	Mean Sqs	F-model	*R* ^2^	*p*-Value
Stress	1	6404	3.635	0.134	**0.001**
Ration	1	3893	2.210	0.082	**0.002**
Stress:Ration	1	2169	1.231	0.045	0.138
Residual	20	1762		0.739	
Total	23			1.000	

*Significant relations (p ≤ 0.05) are displayed in bold.*

Correlations between the bacterial beta-diversity and larval fitness measurements after 96 h exposure to stressed algae, showed significant and strong positive associations between the mortality rate (*r* = 0.52, *p* = 0.001), abnormality rate (*r* = 0.519, *p* = 0.001), bacterial richness (*r* = 0.435, *p* = 0.005) and the stressed treatments. Percent swimming (*r* = 0.269, *p* = 0.033) and feeding (*r* = 0.38, *p* = 0.004) were positively associated with the control samples (Figure 8).

##### Relationships between algal treatment, larval fitness measurements, and specific bacterial genera

**FIGURE 9:**
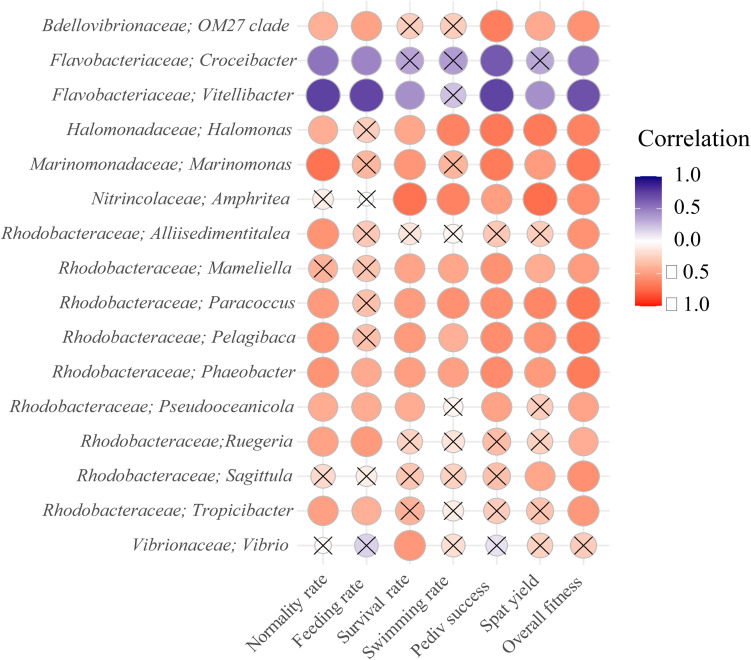
Pearson correlations of bacterial genera with log transformed and normalized oyster fitness metrics after 96 h of exposure. Positive correlations are presented in blue, while negative correlations are presented in red. The overall fitness represents a sum of all fitness metrics. Only the 15 genera with highest absolute correlation with overall fitness are displayed, including the Maribacter and Vibrio genera. Non-significant correlations are marked by a cross sign.

Pearson correlations of the centered-log ratio abundance of bacterial genera with log-transformed fitness measurements showed mostly strong negative relationships for clade OM27 of the *Bdellovibrionaceae* family, the genera *Halomonas, Marinomonas, Amphritea*, *Sneathiella* and several genera of the *Rhodobacteraceae* family such as *Paracoccus, Phaeobacter* and *Rhodovulum* (Figure 9). Although the *Vibrio* genera was also found to have a negative relation with fitness in general, only the correlation with survival rate was found to be significant. In contrast, the genera *Aurantivirga, Croceibacter, Vitellibacter* and *Antarctobacter* of the *Flavobacteriaceae* family, were all positively correlated with larval fitness measurements (Figure 9).

**FIGURE 10:**
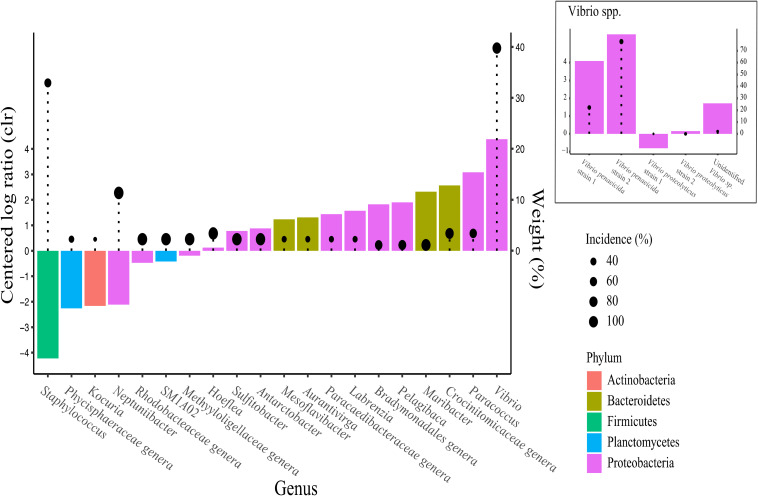
Analysis of composition of microbiomes (ANCOM) of the different taxa between algal feeding ration treatment using the centered log ratio (clr) abundance at genus level. Taxa more abundant under low ration have negative values and vice versa. Weights displayed by lollipops represent the percentage of significant pairwise analyses testing differences in taxa abundance between treatments using each other taxa as denominator. Taxa with higher weights are more likely to be truly affected by the treatment.

Feeding ration was found to affect the bacterial genera and their abundance. *Vibrio, Paracoccus*, an unidentified *Crocinitomicaceae* genera and *Maribacter* were found to be particularly associated with high feed ration while *Staphylococcus*, an unidentified *Phycisphaearaceae, Kocuria* and *Neptuniibacter* genera were more abundant under low feeding conditions (Figure 10). Two different strains of *Vibrio*, *V. penaeicida* and an unidentified *Vibrio* species (Figure 10) were the most abundant/upregulated under high feed conditions.

## Discussion

### Effects of Compromised Algal Feed on the Larval Performances of Oysters

In bivalve hatcheries, there is an expectation that feeding early life stages of bivalves with compromised algal cultures may negatively affect their overall fitness and therefore affect hatchery production outcomes (Kaspar et al., 2014). However, to our knowledge, no study has systematically demonstrated the implications, to developmental success and spat yield, of feeding stressed microalgae to oyster larvae.

Measurements of previously determined indicators of algal health (Rolton et al., 2020) confirmed that cultures of *C. calcitrans* (CC) and *T. lutea* (T-Iso) were effectively stressed by high-pH conditions. Reduced algal density, fluorescence, quantum yield, cell viability, and altered morphology and ROS production measured in this study are in agreement with findings from Rolton et al. (2020) and indicate the compromised health status of these microalgal species fed to oyster larvae. Manipulation of growing conditions (light, temperature, nutrients) have been shown to affect lipid composition of microalgal species like T-Iso (e.g., nitrogen limitation, Da Costa et al., 2017). Likewise, Leal et al. (2021) recently showed how altered culture conditions can affect the production yield and biochemical composition of T-Iso, and subsequently performances of *Saccostrea glomerata* oyster spat.

In this study, high-pH condition resulted in a significant increase (5 times) in neutral lipids in CC as measured by flow-cytometry. This altered lipid profile of the algal feed may be associated with poorer larval outcomes under high ration regime. Indeed, Da Costa et al. (2016) demonstrated that feeding *C. gigas* larvae with a selected strain of T-Iso overabundant in neutral lipids did not improve larval performances. Unlike Da Costa et al. (2016) where growing conditions of algal feed were modified to enhance their nutritional value and subsequently improve larval performance, we chose to intentionally stress algal cultures to create a poor-quality algal diet that will ultimately affect oyster larvae performances. Specifically, we found that feeding stressed algae to larvae for 96 h at a low and high ration impaired their normal development, with more morphological abnormalities (including shell deformities, indented mantle, abnormally extended velum) than in larvae fed unstressed algae. These types of defect are frequently reported in bivalve larvae following acute exposure to heavy metals, pesticides, or organic contaminants (Davis and Hidu, 1969; Beiras and His, 1994; Vignier et al., 2016).

The “Low” and “High” algal rations chosen for this study were based on concentrations required to support maximum growth of *C. gigas* larvae, or that mimic overfeeding conditions, respectively. Although a constant algal background concentration ranging from 20 to 40 cells per μL of T-Iso equivalents has been set as a standard ration used in the hatchery production of oysters (Helm and Bourne, 2004; Rico-Villa et al., 2009), high microalgal backgrounds can have deleterious consequences to larval performance. Uneaten algae may lead to the proliferation of opportunistic pathogenic bacteria by offering increased organic matter and cause mass mortality (Helm and Bourne, 2004). Symptoms typical of bacterial infection include an empty digestive gland, swarming of bacteria around the velum, diminished or cessation of swimming, generalized ciliar paralysis and the presence of necrotic tissues, and usually precede a sudden death event (Tubiash et al., 1965; Jeffries, 1982; Prieur et al., 1990). In our study, more pale larvae (i.e., starved) as well as more inactive, sluggish and ‘spinning’ larvae were found in the treatments fed stressed algae, symptoms indicative of a bacterial infection. The feeding impairments observed in the present study could also be the result of morphological abnormalities as indicated by the strong negative correlation between these two fitness parameters (*r* = −0.52; Supplementary Figure 3). Likewise, abnormal swimming could be the result of deformed shells and abnormal velum. However, larval narcosis manifested as a sluggish behavior or absence of swimming is typically associated with the presence of toxic compounds in the larval environment (Saiz et al., 2009; Vignier et al., 2016). Upon reaching stationary phase, algal cells may become less digestible, have deficient nutrient composition, increased bacterial load, and produce toxic secondary metabolites (Helm and Bourne, 2004; Vidoudez and Pohnert, 2008; Da Costa et al., 2017). Algal metabolites such as polyunsaturated aldehydes and more generally oxylipins are byproducts of lipid peroxidation and have been shown to be produced by some marine diatom species as an activated chemical defense against future generations of potential grazers (Miralto et al., 1999). These oxylipins are known to be cytotoxic and teratogenic to several marine invertebrates (Caldwell, 2009; Ruocco et al., 2016). It is possible therefore that the diatom species used in our study, *C. calcitrans*, could produce similar metabolites under stressful conditions that could have detrimental effects on oyster larvae. Alternatively, some *Vibrio* spp. can produce toxic extracellular compounds that have been implicated as factors of pathogenicity in bivalve oyster larvae (Nottage and Birkbeck, 1987; Hasegawa et al., 2008). The fact that only high rations of stressed algae impaired swimming activity of the larvae, whilst low ration did not, suggests a dose-dependent effect of the compromised algal food and further support our hypothesis of toxicity related to algal and/or bacteria metabolites. Moreover, by increasing the volume of algae to account for the lower cell concentration in the stressed cultures, it is possible that we increased the probability of larval exposure to excess nutrients from the growing media or more importantly algal exudates including toxic extracellular metabolites. In light of this, future research should focus on the potential toxic effects of algal metabolites to early life stages of bivalve. Such studies could provide additional clues as to why microalgal quality, and particularly overfeeding of larvae, can result in sub-optimal larval rearing conditions in the hatchery.

Seven days of rearing oyster larvae in recovery conditions (i.e., standard ration of unstressed algae) did not improve the performances of larvae previously fed high rations of stressed algae. Indeed, the pediveliger success, which corresponds to the net yield of larvae to reach settlement, was over 6 times lower than in the control. Interestingly, overfeeding larvae with a high ration of unstressed algae for 4 days resulted in increased pediveliger success. The benefits of a microalgal diet rich in marine lipids to oyster larvae is well supported in the literature and was shown to support rapid larval growth and promote higher larval competence (Enright et al., 1986; Haws et al., 1993; Powell et al., 2002). We can therefore postulate that high ration of control algae could have contributed to high rates of competent larvae. After settlement, however, similar spat yields were recorded between the two control treatments, underlining that overfeeding is not advantageous in the long term. Furthermore, our results showed that overfeeding 11-day old larvae with compromised algae resulted in spat yields four times lower than in any other treatments. Surprisingly, spat yield in oysters previously fed low rations of stressed algae did not differ from the control treatments. The significantly higher levels of neutral lipids measured in the stressed CC could partly explain this result as neutral lipids such as Triacylglycerols (TAG) are particularly important to support the metamorphosis of oysters (Chu and Webb, 1984; Ben Kheder et al., 2010). However, an excess of TAG supplied to larvae under a high ration feeding regime may disturb larval metabolism by creating an imbalance between energy reserves and membrane structures in the larvae, and could produce deleterious effects (Pernet et al., 2004; Da Costa et al., 2016).

### Characterization and Comparison of the Microbial Communities Associated With Algae, Larvae, and Seawater

Apart from Arfken et al. (2021) who described the microbiome associated with different developmental larval stages of another species of oyster, *Crassostrea virginica*, raised in different hatcheries, limited knowledge exists on the development of the microbiome in Pacific oysters for a controlled hatchery environment, particularly during their early larval development when microalgal diets of varying quality are provided. Inducing stressful culture conditions allowed us to alter the bacterial composition of the algal food and subsequently investigate the effect on overall survival of oyster larvae.

Compositional analysis of the bacterial communities showed similarities at the phylum level between the three investigated compartments (microalgal feed, larvae, and rearing seawater), with *Proteobacteria* and *Bacteroidetes* being the predominant taxa. Although no discernible effect of the pH stress treatment could be observed at this taxonomic level, the relative abundance of specific phyla varied between the days of exposure, especially for the larvae. Other studies have found that bacterial communities present in oyster hatcheries shared many of the most dominant phyla (e.g., *Proteobacteria, Bacteroidetes*) despite strong temporal changes in relative abundance (Asmani et al., 2016; Stevick et al., 2019). Previous work from Laroche et al. (2018), in which the dynamics of functionally critical bacterial taxa and their successive roles in larval development of Pacific oysters were studied, also found that microbiome composition was primarily affected by temporal changes in the water source. Factors such as temperature, salinity, oxygen content, nutrients, presence of anthropogenic chemicals, or seawater treatments such as UV sterilization potentially influence the microbiome of the seawater in hatchery systems (Fuhrman et al., 2006; Kirchman et al., 2010; Laroche et al., 2018). The increasingly complex bacterial community structure observed in oyster larvae throughout larval rearing is also believed to be driven by the microbiome associated with the microalgal food (Nicolas et al., 2004; Asmani et al., 2016; Laroche et al., 2018). The current study confirms the potential influence of different feeding regimes and health conditions of microalgae to the microbial community turnover in larvae.

The rearing seawater compartment showed the highest bacterial richness compared with the algal food and larval compartments, regardless of the feeding regime (Figure 6), a finding in agreement with several recent studies. Arfken et al. (2021) observed a clear distinction between the larval microbiome of *Crassostrea virginica* and the microbiome of the corresponding rearing seawater. They found a strong negative correlation between nutrient composition and bacterial richness in the seawater but no correlation with the oyster larvae microbiome. When evaluating the performance of *C. gigas* larvae reared in a flow-through system vs. a recirculated system, Asmani et al. (2016) also showed a clear difference between the microbiome present in the rearing seawater and the larval microbiome. Findings from both studies suggest that hatchery water treatment, rearing systems or other husbandry practices may greatly influence the seawater microbiome but may not be as important to the bacterial composition of the larvae. Likewise, Stevick et al. (2019) demonstrated that *C. virginica* larval microbiome (beta-diversity) significantly differed from the microbiome of their corresponding seawater. These results suggest that oyster larvae have a dynamically developing microbiome that can be selectively colonized by transient distinct bacterial taxa. When larvae are fed with stressed algae, their health seems to be compromised and we postulate that larvae are no longer able to keep a strict control over their microbiome, which may explain the increased richness observed in the present study. This is further supported by the finding that bacterial richness in algae under stress, particularly CC, is not increased. In contrast, healthy larvae may be able to exercise selective pressure on their microbiome, explaining the lower host-microbiome diversity compared to the surrounding environment.

### Relationships Between Microbiota Features and Larval Fitness Measurements

The bacterial richness characterized in oyster larvae showed a strong correlation with larval fitness parameters. More specifically, rates of larval abnormality and reduced survival to the pediveliger stage were strongly positively correlated with bacterial richness in larval tissue. A strong clustering of each algal treatment was denoted, with developmental impairments (e.g., mortality and abnormality rates) closely associated with the “Stress High” group, whereas feeding and swimming abilities were positively associated with the “Control” groups (Figure 8). This is a significant finding as it provides an important step toward understanding the potential complex relationship between larval microbial communities and observed larval performance when reared in the hatchery under different culture conditions.

Furthermore, our study revealed for the first time that certain bacterial features (e.g., *Halomonas, Marinomonas, Paracoccus*, or *Phaeobacter*) were clearly associated with poor larval fitness measurements. The ubiquitous species, *Halomonas*, is known to colonize surfaces and produce inhibitory and antimicrobial compounds that prevent competitors from succeeding. During a mass mortality event in a scallop hatchery, Rojas et al. (2009) isolated a strain of *Halomonas* from the biofilm present at the bottom of a larval rearing tank. After incubating scallop larvae for 24 h with that exopolysaccharide (EPS) slime-producing strain of *Halomonas*, pathogenic action was evidenced by agglutination and loss of motility of the larvae (Rojas et al., 2009). *Marinomonas* also appears to be significantly correlated with poor fitness indicators (Figure 9). De Lorgeril et al. (2018) reported that, following OsHV-1 viral burst, *Marinomonas* was one of several bacterial genera involved in the observed septicemia in dying Pacific oysters. Interestingly, *Marinomonas* was only found in the larvae compartment (see Supplementary Figure 1). An increase of this taxa in unhealthy larvae may indicate an imbalance of the larvae microbiome. Both *Halomonas* and *Marinomonas* spp. were detected in scallop larvae and hatchery effluents in Chile: they were found to possess antimicrobial resistant genes (floR; Miranda et al., 2015). In contrast to *Halomonas* or *Marinomonas*, genera from the *Flavobacteriaceae* family (e.g., *Aurantivirga*, *Croceibacter*, *Vitellibacter*, and *Antarctobacter*) appear to be beneficial to oyster larvae and show strong positive relationships with larval performance. Isolates of *Antarctobacter* have been shown to have antibacterial properties against fish pathogens (*Vibrio anguillarum*, Sharifah and Eguchi, 2011), and the use of *Antarctobacter* should be further considered as a potential probiotic in shellfish hatchery.

Collectively, our findings emphasized the importance of the oyster microbiome to successful larval rearing and its putative functional role in healthy larvae. For example, slime-producing strains from the *Halomonas* genus should be closely monitored as they were strongly associated with larvae with deteriorating health, whereas other strains from the *Flavobacteriaceae* family could be beneficial and necessary to ensure normal development.

Members of the *Vibrio* genus are ubiquitous in the marine environment and often associated with (or blamed for) disease and mass mortality events of bivalve larvae (Elston and Leibovitz, 1980; Prado et al., 2005; Rojas et al., 2016). This study found that *Vibrio* spp. were poorly correlated with most of the fitness measurements (Figure 9) compared with other bacterial genera previously described. *Vibrio* spp. were only correlated with mortality rates after 96 h, suggesting secondary infection which may be expected as these bacteria tend to be opportunistic and generally abundant in moribund/dead oysters (Riquelme et al., 1996; Lacoste et al., 2001; De Lorgeril et al., 2018). We observed relatively low levels (5.5%) of *Vibrio* spp. associated with the larvae. This is not unsurprising as our algae-stress was not designed to encourage proliferation of (specific) pathogens. The presence of predatory bacteria such as *Bdellovibrionaceae*, which can attack and rupture a large variety of gram-negative bacteria, may also have limited the proliferation of pathogenic taxa such as *Vibrio* spp. (Cao et al., 2019). Nonetheless, *Vibrio* consisting of the two different strains of *V. penaeicida* and an unidentified *Vibrio* species (Figure 10), were strongly associated with high feed rations, regardless of stress. *Vibrio penaeicida* is known to produce exotoxins that are pathogenic to shrimp larval stages (Aguirre-Guzmán et al., 2001) and we could speculate these may also be harmful to early life stage of oysters.

Our findings underline the ubiquity of *Vibrio* spp. in the marine environment and that *Vibrio* can be associated with both healthy oysters and mass mortality events. Regardless of whether *Vibrio* has a causative or associative relationship with larval mortalities, our results indicate that overfeeding can be a source of *Vibrio* to the larval culture and potentially increase the risk of infection via pathogenic strains when present. Understanding the positive and negative effects of specific bacteria on early shellfish life stages is a complex and ongoing challenge. Monitoring microbial communities during actual pathogenic infections will provide a powerful hypothesis generation tool for more targeted (e.g., Koch’s postulate) methodologies to definitively resolve specific bacterial virulence and will improve our ability to predict mortality events in aquaculture and hatchery systems.

To conclude, the present study systematically demonstrated that feeding compromised microalgae to developing oyster larvae resulted in reduced larval development and survival. This highlights the importance of feeding healthy cultures of microalgae, at the appropriate ration, to developing larvae of Pacific oysters. Overfeeding larvae with compromised algae for 96 h reduced their overall health by increasing deformities, reducing feeding efficiency and swimming ability, altering larval growth, and ultimately leading to death. Even after recovery under normal feeding conditions, long-lasting effects were observed with fewer larvae reaching the pediveliger stage and becoming spat. Nonetheless, we showed that it is possible to alleviate the detrimental long-term effects of a poor algal batch on oysters by reducing the quantities fed to the larvae at any given point in their development. In other words, it might be okay to feed larvae with sub-optimal quality algae at reduced quantities and for a short period of time. Correlation analyses also showed that swimming activity was a good proxy to predict a successful spat outcome and could potentially represent a useful early indicator of larval stress.

Bacterial characterization using metabarcoding gave valuable insights into the dynamics of the microbiome associated with larvae. For instance, higher bacterial richness was found in the rearing seawater compared to the larvae and the microalgae compartments, regardless of the feeding regime. Bacterial richness in larval tissue was higher in stressed, high-feed treatments, and strongly negatively correlated with larval performance measurements. The association between specific microbiota features (e.g., bacterial richness) and larval performance identifies the opportunity for such features to be explored as proxies for compromised oyster larval health and suggests a hypothetical functional role in maintaining larval health. Finally, bacterial profiling confirmed that feeding algae of sub-optimal quality in large quantities could affect larval performance, subsequently decreasing spat yield, and was associated with shifting bacterial community composition (dysbiosis) including increased abundance of potentially pathogenic strains of bacteria (including, but not limited to, *Vibrio* strains).

Characterization of differences in the bacterial community associated with Pacific oyster larvae in a controlled hatchery environment is an initial step toward understanding the role and function of the microbiome on the phenotype of oysters. Further experimental studies are needed to determine the causative (or otherwise) nature of these associations, and in particular the implications of genetics on both the role, and control, of the microbiota using a reduced set of performance measures. Future hatchery application of such approaches to screen for pathogens and assess health status of microalgal food and larvae may lead to improved hatchery efficiency and reliability.

## Data Availability Statement

The datasets presented in this study can be found in the NCBI online repository (www.ncbi.nlm.nih.gov/) under Project No. PRJNA729567. The accession numbers are: SRA18230711 to SRA18230712.

## Author Contributions

JV, AR, XP, and NK: conception and design. JV, AR, PW, and KK: data collection and performance hatchery trials. PW, KK, and BT: collection and preparation of samples for sequencing. OL: bioinformatic analyses. JV, AR, and OL: writing. All authors: revision/editing.

## Conflict of Interest

PW was employed by company Moana New Zealand Ltd. The remaining authors declare that the research was conducted in the absence of any commercial or financial relationships that could be construed as a potential conflict of interest.

## Publisher’s Note

All claims expressed in this article are solely those of the authors and do not necessarily represent those of their affiliated organizations, or those of the publisher, the editors and the reviewers. Any product that may be evaluated in this article, or claim that may be made by its manufacturer, is not guaranteed or endorsed by the publisher.
