# Supplementary material for: Dietary Exposure of Pacific Oyster (Crassostrea gigas) Larvae to Compromised Microalgae Results in Impaired Fitness and Microbiome Shift
Source: Front Microbiol. 2021 Aug 24;12:706214. doi: 10.3389/fmicb.2021.706214 (PMC8421776; doi:10.3389/fmicb.2021.706214)
Supplement: Supplementary file 1 [file Data_Sheet_1.docx]

Supplementary figures and tables


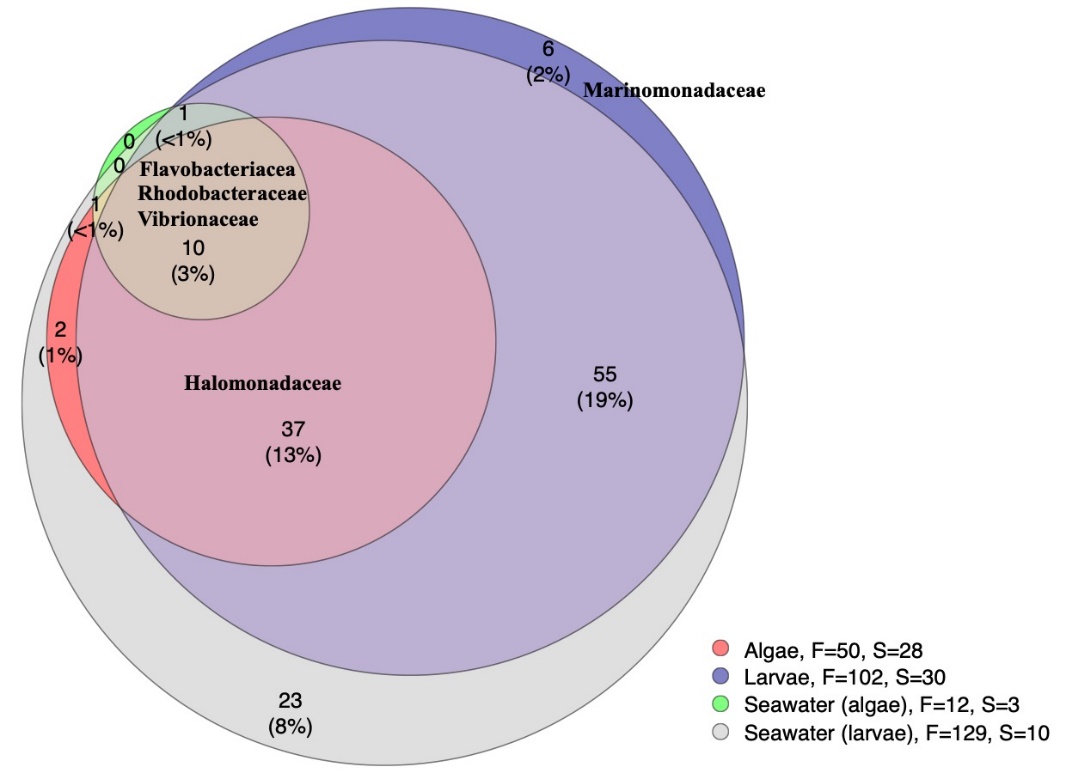


Figure S1. Venn diagram of bacterial families shared between the larval rearing seawater (grey), the larval tissue at day 15PF / day 4 of exposure (purple) and the microalgal feed (combined CC and T-Iso, in red). The numbers in the fields, also expressed in %, describe the number/percent of taxa characterized per dataset. F = total number of unique families, S = number of samples. The presence of few families of particular interest are overlaid in their respective compartments.

Figure S2. Boxplots of amplicon sequence variant (ASV) bacterial richness characterised between control (blue) and stress (red) treatments for *Chaetoceros calcitrans*, CC (Top) and *Tisochrysis lutea*, Tiso (bottom).

Figure S3. Correlation chart of log transformed bacterial richness and fitness metrics. The bottom of the diagonal display bivariate scatterplots with a fitted line. The top of the diagonal display values of the correlation with significance indicated as stars while the in-between panels show histograms of value distribution. p-values (0.001, 0.01, 0.05, 0.1) <=> symbols (“***”, “**”, “*”, “.”). Transf.Abn.rate = log-transformed abnormality rate, Transf.Pct.swimming = log-transformed swimming percentage, Transf.Feeding = log-transformed feeding percentage, Transf.morta.rate.D4 = log-transformed mortality rate at day 4, LogRichness = log-transformed bacterial richness, Transf.pediv.success = log-transformed pediveliger success.

Table S1. Permutational analysis of variance in larval microbiome diversity between treatment conditions nested in days of exposure, including interaction between treatments, using 999 permutations. Significant relations (*p ≤ 0.05*) are displayed in bold.

| Terms | Df | Mean Sqs | F-model | R^2^ | p.value |
| --- | --- | --- | --- | --- | --- |
| Days of exposure | 1 | 8887 | 5.066 | 0.136 | **0.001** |
| Days of exposure:Stress | 1 | 6447 | 3.675 | 0.099 | **0.001** |
| Days of exposure:Ration | 1 | 3918 | 2.233 | 0.06 | **0.005** |
| Days of exposure:Stress:Ration | 1 | 2192 | 1.230 | 0.033 | **0.139** |
| Residual | 25 |  |  | 0.672 |  |
| Total | 29 |  |  | 1.000 |  |
